# Supplementary figures and images for: Functional characterization of 5′ UTR cis-acting sequence elements that modulate translational efficiency in Plasmodium falciparum and humans
Source: Malar J. 2022 Jan 6;21:15. doi: 10.1186/s12936-021-04024-2 (PMC8739713; doi:10.1186/s12936-021-04024-2)

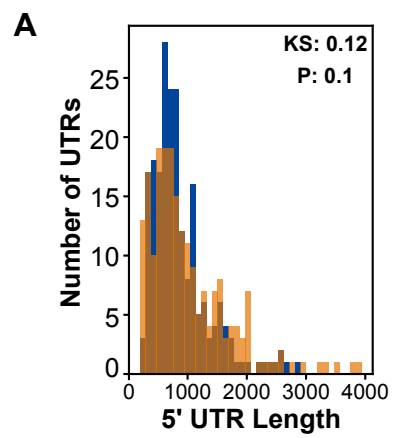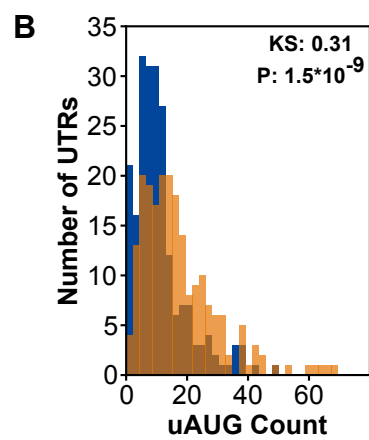

Supplement: Supplementary file 6 — Additional file 6: Fig. S1. Further comparison of 5′ UTR features of genes in the bottom 10% and top 10% of TEs in the late trophozoite stage using data from Caro et al. [18]. a) Distributions of the 5′ UTR lengths from genes with high (blue) or low (yellow) TE. KS test statistic comparison of the two: 0.12 p-value 0.1. b) Distributions of the total number of uAUGs in the 5′ UTRs from genes with high (blue) or low (yellow) TE. KS test statistic comparison of the two: 0.31 p-value 1.5*10−5. [file 12936_2021_4024_MOESM6_ESM.pdf]

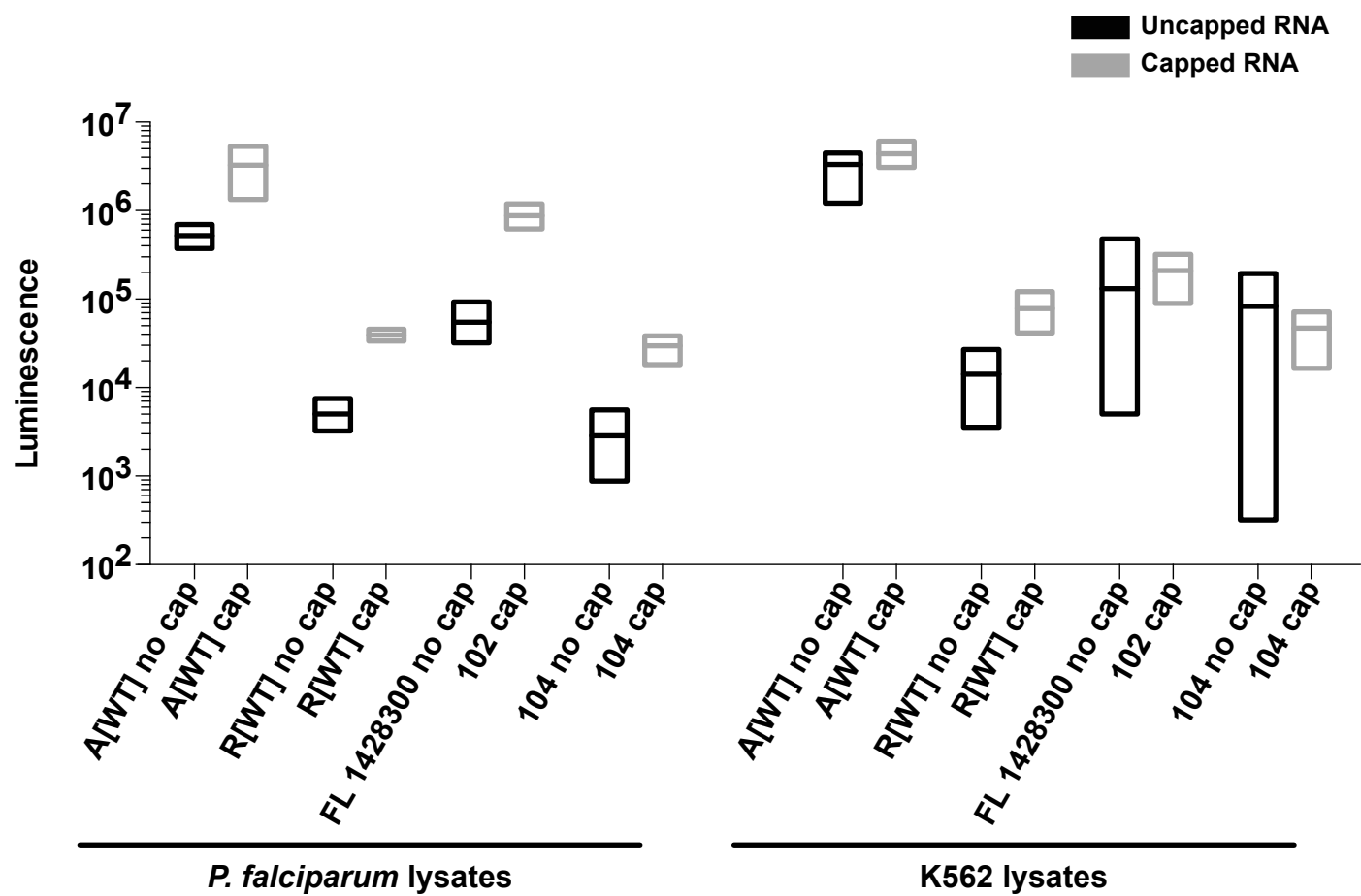

Supplement: Supplementary file 7 — Additional file 7: Fig. S2. The raw luminescence signal from capped and uncapped RNAs in P. falciparum and K562 in vitro translation. [file 12936_2021_4024_MOESM7_ESM.pdf]

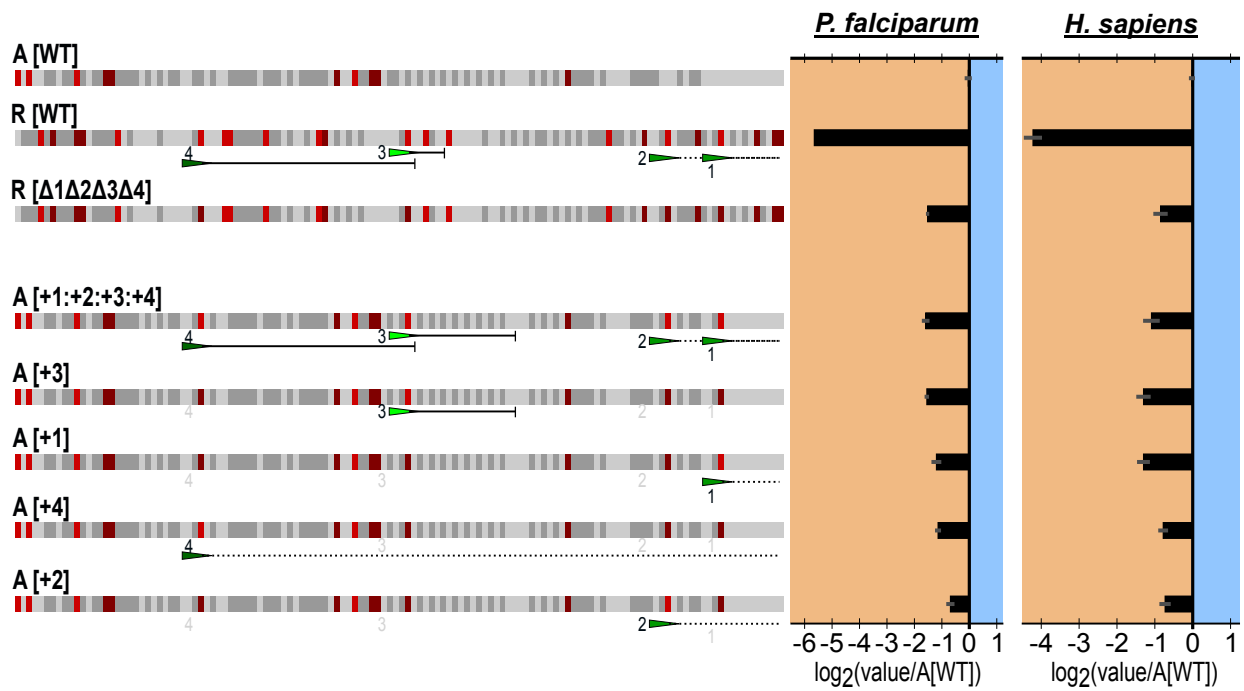

Supplement: Supplementary file 8 — Additional file 8: Fig. S3. The 4 uAUGs from R[WT] exchanged into A[WT] at the same positions showing that the repressive effect is conferrable to other contexts. Graphed for each is the average and SEM of log2(each experimental value normalized to the experimental average of A[WT]). [file 12936_2021_4024_MOESM8_ESM.pdf]

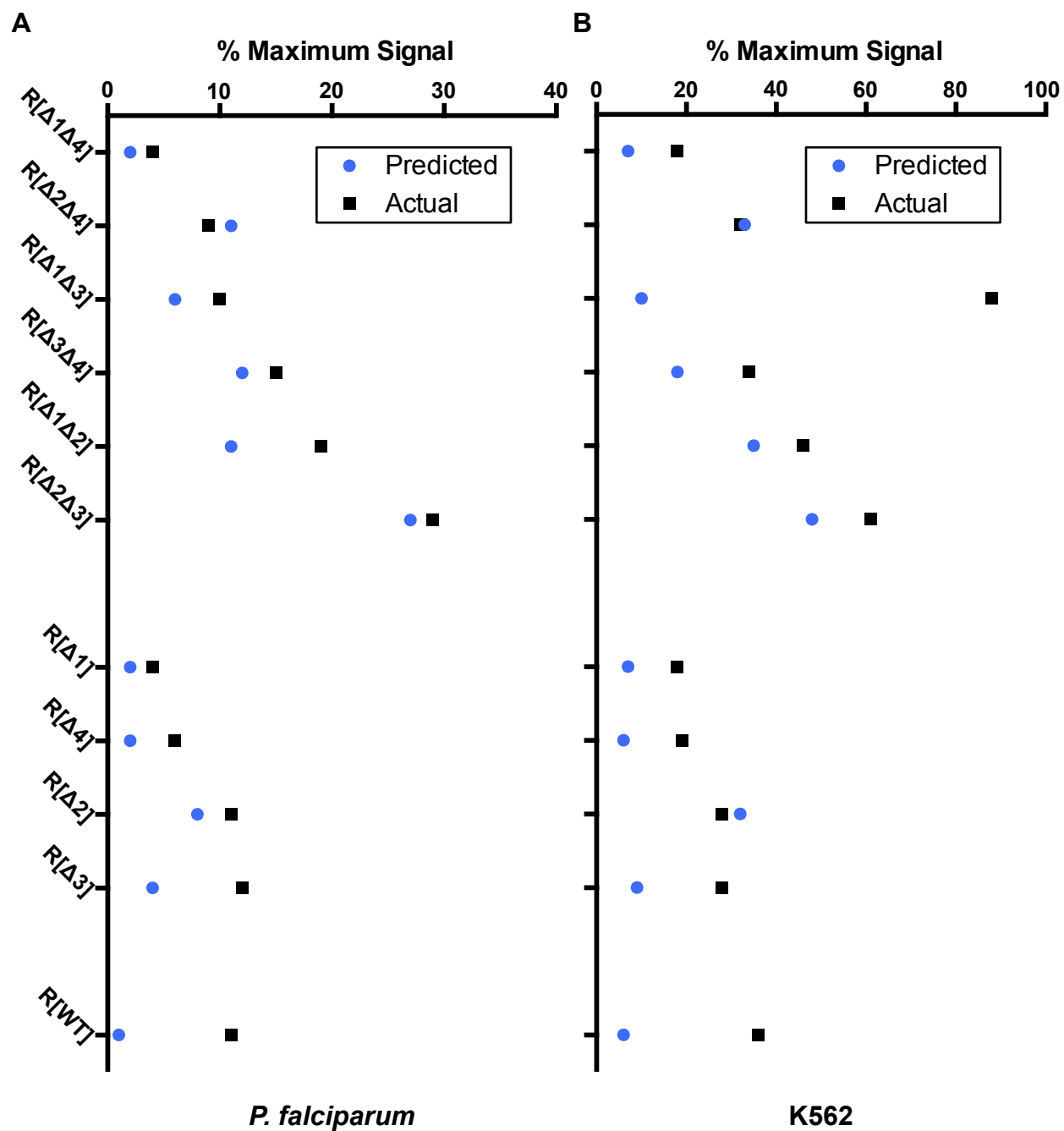

Supplement: Supplementary file 9 — Additional file 9: Fig. S4. Predicted repressive effect of combinations of the uAUGs in R[WT] based on their individual activities for (a) P. falciparum and (b) K562. [file 12936_2021_4024_MOESM9_ESM.pdf]

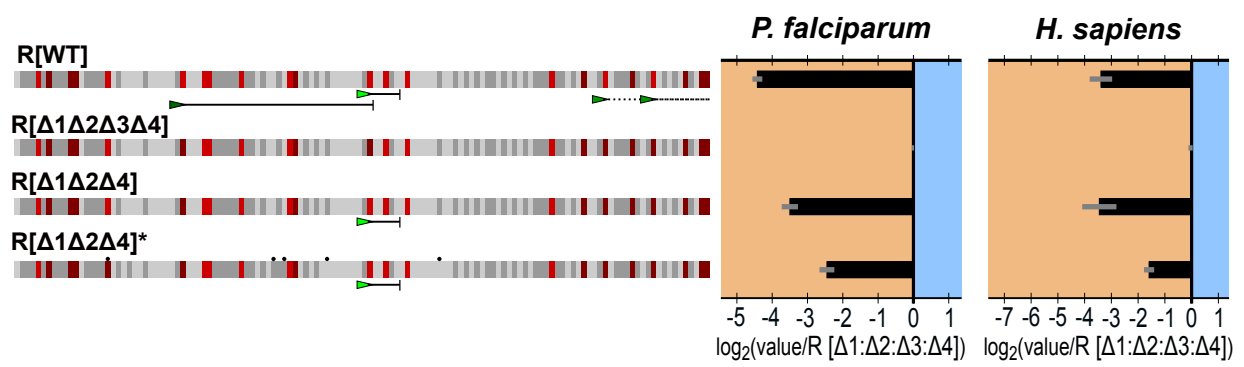

Supplement: Supplementary file 10 — Additional file 10: Fig. S5. To eliminate all downstream stop sites for moving the out of frame non-terminated uAUG, 6-point mutations had to be added to the 5′ UTR. R[Δ1:Δ2:Δ4]* was made with those point mutations to compare to R[Δ1:Δ2:Δ4]. Graphed for each is the average and SEM of log2(each triplicate value/ average of R[Δ1:Δ2:Δ3:Δ4] experimental triplicates). [file 12936_2021_4024_MOESM10_ESM.pdf]

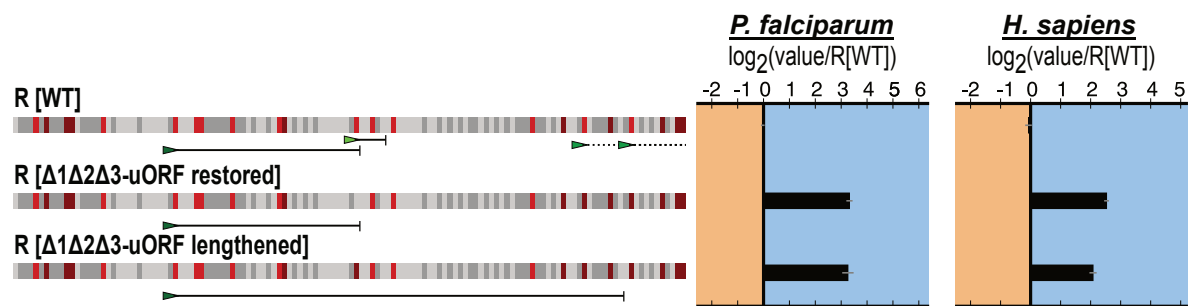

Supplement: Supplementary file 12 — Additional file 12: Fig. S7. The effect of lengthening the uORF from uAUG-3. Graphed for each is the average and SEM of log2(each triplicate value/ average of R[Δ1:Δ2:Δ3:Δ4] experimental triplicates). [file 12936_2021_4024_MOESM12_ESM.pdf]
