# Supplementary material for: Functional characterization of 5′ UTR cis-acting sequence elements that modulate translational efficiency in Plasmodium falciparum and humans
Source: Malar J. 2022 Jan 6;21:15. doi: 10.1186/s12936-021-04024-2 (PMC8739713; doi:10.1186/s12936-021-04024-2)
Supplement: Supplementary file 11 — Additional file 11: Fig. S6. The predicted free energy of the secondary structure with in a 30-nucleotide sliding window moved by 1 nucleotide across the 5′ UTRs used to evaluate the effect of GC content. [file 12936_2021_4024_MOESM11_ESM.pdf]

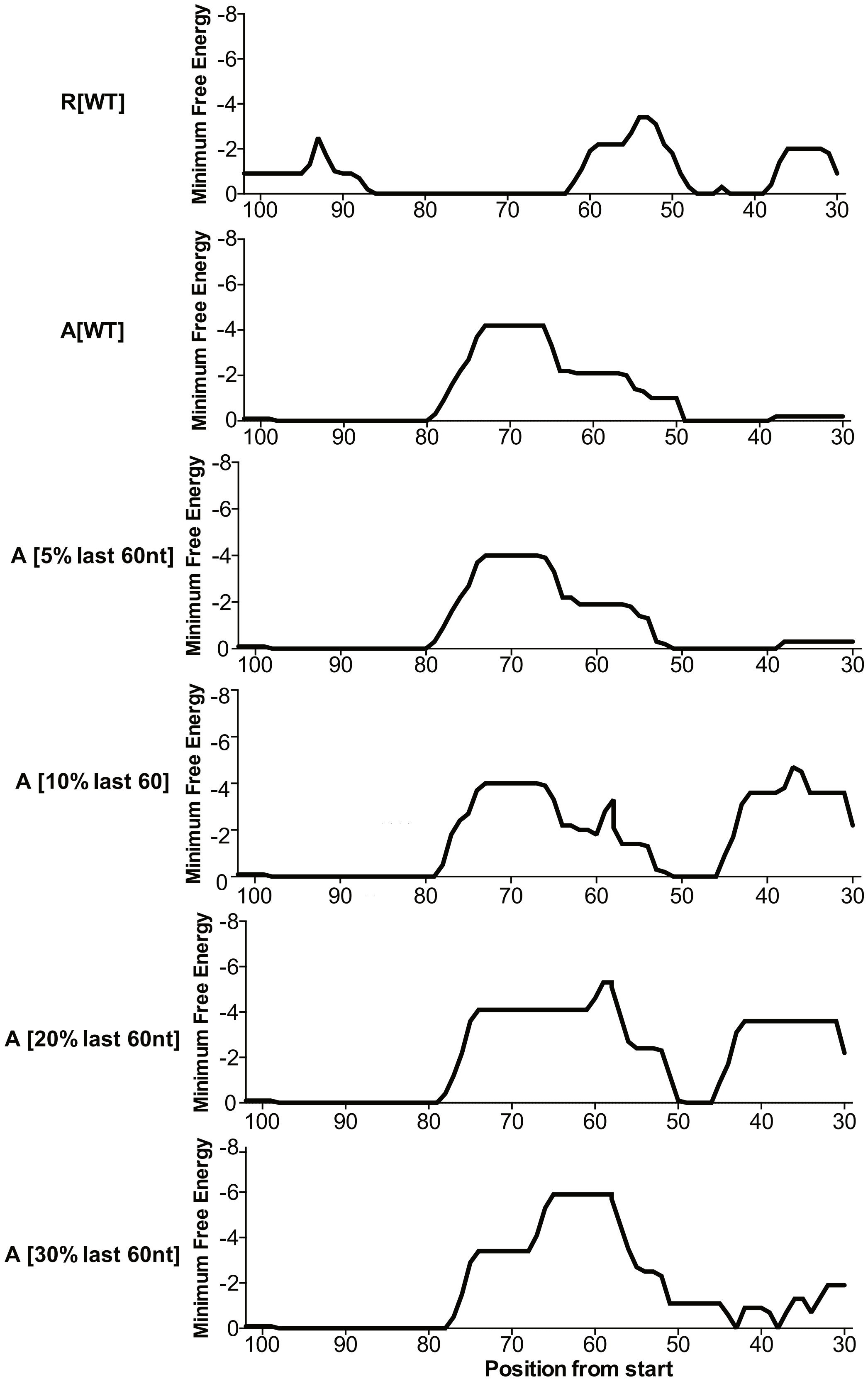

Supplemental figure 5: The predicted free energy of the secondary structure with in a 30-nucleotide sliding window moved by 1 nucleotide across the 5' UTRs used to evaluate the effect of GC content.
